# Public health insurance and enrollees’ diet structure in rural China

**DOI:** 10.1016/j.heliyon.2022.e09382

**Published:** 2022-05-09

**Authors:** Qihui Chen, Chunchen Pei, Juerong Huang, Guoqiang Tian

**Affiliations:** a Beijing Food Safety Policy & Strategy Research Base, China Agricultural University, PR China; b Research Center for Future Education, School of Economics and Resource Management, Beijing Normal University, PR China; c College of Economics and Management, China Agricultural University, Beijing, PR China; d Department of Finance, College of Economics and Management, China Agricultural University, PR China

**Keywords:** Public health insurance, Diet diversity, Diet balance, Difference-in-differences, Rural China

## Abstract

This study examines the consumption-stimulation function of public health insurance (PHI) programs from the perspective of food consumption. We estimate the impact of enrollment in rural China's PHI program, the New Cooperative Medical Scheme (NCMS), on the insured's diet diversity and diet balance using panel data from the China Health and Nutrition Survey. Exploiting temporal and spatial variations in the program's local implementation, our difference-in-differences estimation (combined with propensity score matching in some analyses) reveals significant increases in the insured's diet diversity, overall diet balance, and nutrition intakes. However, the program's consumption-stimulation function is not entirely beneficial. While NCMS enrollment reduced the incidence of under-consumption of animal products and fruits, it raised that of over-consumption of grains, imposing potential health risks on the insured.

## 1 Introduction

Many developing countries have implemented public health insurance (PHI) programs to promote population health in the past few decades. China is no exception. In 2003, China launched the PHI program serving its rural population, the New Cooperative Medical Scheme (NCMS), aiming to revive its rural health insurance sector. The predecessor of the NCMS, the Cooperative Medical Scheme (CMS), was implemented in the 1950s. Mainly financed by village-level funding sources, the CMS operated health stations, paid village doctors, and provided medicine to its enrollees, making primary healthcare accessible to farmers and offering them financial protection against large medical expenses. On the eve of China's rural reforms in 1978, the CMS covered about 90% of China's rural population (Yip and Hsiao, 2008). However, CMS coverage plummeted as China's rural communes collapsed in the reform era. Throughout the subsequent two decades, health insurance coverage never exceeded 20% of China's rural population, substantially raising rural residents' financial and health risks (Yip and Hsiao, 2008). In response, the Chinese government launched the NCMS, operating this new system based on three principles, i.e., voluntary participation, county-level administration, and a focus on catastrophic illness (inpatient services), with funding from both the government and the enrollees.^1^ It has been found that enrollment in the NCMS has led to more frequent healthcare utilization (Qin et al., 2014) and better health outcomes among its enrollees (Chen and Chu, 2019; Fan et al., 2019; Chen et al., 2020).

While the ultimate goal of PHI programs is to improve population health, they may also serve to stimulate non-medical consumption. For example, health insurance may reduce the uncertainty about future medical expenditures faced by the insured and (thus) their precautionary savings (Cheung and Padieu, 2015). The resulting increase in disposable income will allow the insured to increase their consumption in non-medical dimensions. In fact, the NCMS explicitly sets consumption stimulation as an essential target (Bai and Wu 2014). Fulfilling its consumption-stimulating role, the NCMS significantly raised the insured's daily (Zhao, 2019), durable (Cai et al., 2016), and food consumption (Ma and Zhang, 2011). However, when it comes to food consumption, more is not always better. In particular, over-consumption of certain foods, especially those high in fat, sugar, and sodium, imposes severe threats to consumers' health (GBD 2017 Diet Collaborators, 2019). It is thus necessary to examine whether PHI programs help improve the insured's diet structure (e.g., diet diversity and balance) *beyond* increasing their food intakes. Unfortunately, few have assessed the impacts of the NCMS, China's rural PHI program, in this regard.

The lack of attention paid to the insured's diet structure, a crucial aspect of the quality of food consumption, when examining PHI programs' consumption-stimulation function is unfortunate, especially in developing-country settings. The Food and Agriculture Organization of the United Nations (FAO) revised the definition of food security in 1996, placing more emphasis on the *quality* aspect of food security: Food security does not merely refer to access to sufficient food quantity but also embodies access to food with high *quality*, i.e., “safe and nutritious food which meets individuals' dietary needs and food preferences for an active and healthy life” (FAO, 1996). This revision reflects an updated understanding of a pressing issue facing the developing world. Even in countries that have achieved the quantity aspect of food security, inadequate intakes of essential nutrients, such as vitamins and minerals, have caused malnutrition and weakness in millions of people residing there (Skoufias et al., 2009). The imbalanced diet structure may, in turn, undermine their labor productivity and even their countries' future development (Jha et al., 2009; Salois, 2012). If PHI programs can improve the insured's diet structure, expanding the coverage of these programs and strengthening their functionality would certainly be desirable.

Rural China provides an interesting case to study. Its residents have traditionally dwelled on a monotonous diet consisting primarily of grains and few other nutrients (Chang and Wang, 2016). As the Chinese economy developed quickly in recent decades, rural Chinese residents' diet quality and structure have also greatly improved.^2^ However, it remains unclear to what extent these improvements can be attributed to the implementation of the NCMS, China's rural PHI program. The present study attempts to provide an answer by analyzing a longitudinal dataset from the China Health and Nutrition Survey (CHNS).

Exploiting temporal and spatial variations in the NCMS's local implementation, we adopt a difference-in-difference (DID) approach (combined with propensity score matching in some analyses) to estimate the impacts of program enrollment on the insured's diet structure. A unique feature of the CHNS data, i.e., detailed information on each sampled individual's consumption of more than 1,500 food items recorded during three consecutive days, allows us to comprehensively assess rural Chinese residents' diet structure. Specifically, we used this information to construct two diet-structure indicators (with several sub-indicators), with reference to indicators that are widely used in the diet-quality literature, such as the Healthy Eating Index and the Diet Quality Index (Kennedy et al., 1995; Patterson et al., 1994; Reedy et al., 2018). The first indicator we constructed, the diet-diversity score (DDS), is a “variety-based” indicator that counts the number of food groups one consumes out of the set of foods recommended by the Chinese Nutrition Association (CNA, 2008). The second indicator, the diet-balance index (DBI), is more comprehensive and “balance-based”, which takes into account not only one's diet diversity but also one's under- and over-consumption of foods.

Our DID estimation based on a sample of 2,260 rural Chinese individuals of working age found that enrollment in the NCMS increased the insured's diet diversity in a statistically significant manner, but this effect is *not* entirely beneficial. More specifically, the effects on the DBI reveal that while NCMS enrollment significantly reduced the incidence of under-consumption of some foods (e.g., animal products and fruits), it also significantly raised the incidence of *over-consumption* of some other foods (e.g., grains). On a more positive note, NCMS enrollment was found to *reduce* the insured's consumption of cooking oil and salt, revealing a health-promoting channel that does not work through lowering the insured's precautionary savings. In contrast, the DDS, despite its recent popularity in the nutrition and food economics literature (Chen et al., 2017; Kassie et al., 2020; Quisumbing et al., 2021; Santoso et al., 2021), fails to capture the over-eating effect and reductions in oil and salt intakes owing to NCMS implementation. Nor can the commonly-used nutrition-intake measures (Chen and Chu, 2019; Liang and Gibson, 2018; Liu, 2016; Ma and Zhang, 2011; Zhao et al., 2013).

With these analyses, our study contributes to the literature in three ways. First of all, this study provides new evidence from rural China that even though PHI programs *can* improve the insured's diet in many dimensions, the food consumption-stimulation function of these programs may raise the risk of over-eating among the insured. Secondly, our findings complement previous findings on the impacts of the NCMS, China's rural PHI program, in non-medical dimensions, helping to depict a fuller picture of this program's impacts. Finally, not only did we carefully construct two diet-structure indicators (one variety-based and one balance-based) to assess Chinese residents' diet quality, but we also compared the relative performance of these indicators. The recent decade has witnessed increasing applications of diet-structure indicators (—see the references above), but most of these indicators are variety-based rather than balance-based. Using the expansion of the NCMS as a policy experiment, we discovered that the (quick and low-cost) variety-based indicator (DDS) fails to detect important program impacts that the balance-based indicator (DBI) managed to capture.

The remainder of this paper proceeds as follows. The next section describes the data. Section 3 develops our empirical models. Section 4 reports and discusses our findings. The final section concludes and points out several directions for future research.

## 2 Data

### 2.1 Survey and sampling

The China Health and Nutrition Survey (https://www.cpc.unc.edu/projects/china) is an ongoing project conducted by a collaborative effort between the Caroline Population Center at the University of North Carolina and the National Institute of Nutrition and Food Safety at the Chinese Center for Disease Control and Prevention. The main survey covers nine provinces: Guangxi, Guizhou, Heilongjiang, Henan, Hubei, Hunan, Jiangsu, Liaoning, and Shandong (—see the CHNS website for the locations of these provinces: https://www.cpc.unc.edu/projects/china/about/proj_desc/chinamap). These provinces vary substantially in geographical, social, and economic characteristics. Strictly speaking, these provinces are not representative of China as a whole. But the CHNS data are by far the most widely-used data source for studying food consumption, nutrition, and health issues in China.^3^

A multistage, random sampling procedure was adopted in 1989 by the CHNS project team to select target households. More specifically, counties in each project province were first stratified by income levels (low, middle, and high). Then, a weighted sampling scheme was used to randomly select four counties from each province. In addition, the provincial capital and a lower-income city were selected whenever feasible—in two provinces, a large city other than the provincial capital was selected. Villages and townships within the selected counties and urban/suburban communities within the selected cities were chosen randomly. Follow-up surveys were conducted in 1991, 1993, 1997, 2000, 2004, 2006, 2009, 2011, 2013, and 2015. Approximately 4,400 households and 25,000 individuals have participated in the CHNS. Our primary analysis uses two consecutive waves of data collected in 2004 and 2006; data collected in 2000 are also used to perform falsification tests. The primary reason for focusing on this time window is that NCMS coverage had reached 95% of China's rural counties by 2009 (Liu, 2016), leaving no suitable control group in the waves conducted after 2006.

We further applied several restrictions to form the analytical sample. First, since only individuals with a rural permanent residential permit (*Hukou*) are eligible for NCMS enrolment, we excluded urban *Hukou* holders from the sample. Second, we restricted our attention to rural *Hukou* holders who were of working age (18–60 years) in 2004. We excluded children under age 18 to ensure the accuracy of food-intake information, as they may not accurately recall the food items consumed in the past three days. In many areas in rural China, school-age children are also covered by school-based insurance programs, leaving very few observations for assessing the effects of NCMS enrollment. Individuals over 60 were excluded because they may not be in the labor force at the time of the survey. Since one of our diet-structure indicators, the diet-balance index (detailed in the next section), is defined based on one's daily activity level, including individuals of retirement age may obscure the health insurance-food consumption relationship. Finally, to avoid confounding the impacts of the NCMS with those of other health insurance programs (e.g., commercial insurance and health insurance for women and children), individuals enrolled in other health insurance programs were excluded from the analysis. These restrictions resulted in an analytical sample of 2,260 individuals and 6,343 individual-wave observations.

As shown in panel B of Table 1, the vast majority of individuals in the analytical sample are ethnic Han. Slightly more than half are female. At the baseline of our study (2004), the average individual was 43.8 years old, married (with two children), healthy (with almost no chronic health conditions), and had completed 6.6 years of formal education. Nearly all individuals (94%) were working, with a per capita household income of roughly 4,200 yuan (≈508 U.S. dollars in 2003).

**Table 1:** Summary statistics of variables observed at the baseline (2004).

Sample	Mean	SD	Mean	SD	Mean	SD
	All		Participants		Nonparticipants	
*A. Dietary outcomes*						
Diet-diversity score (DDS)	4.38	[1.02]	4.49	[0.97]	4.29	[1.05]
Diet-balance index (DBI)	-21.11	[7.93]	-20.37	[7.41]	-21.73	[8.29]
DBI-O (over-consumption)	13.10	[5.10]	13.61	[4.89]	12.68	[5.23]
DBI-U (under-consumption)	-34.21	[4.45]	-33.96	[4.27]	-34.42	[4.58]
DBI-distance (=|DBI-O| + |DBI-U|)	43.71	[4.74]	43.57	[5.01]	43.65	[4.86]
Log(calorie)	7.71	[0.31]	7.71	[0.29]	7.71	[0.33]
Log(carbohydrate)	5.85	[0.33]	5.83	[0.32]	5.87	[0.33]
Log(protein)	4.11	[0.35]	4.12	[0.35]	4.10	[0.35]
Log(fat)	4.03	[0.58]	4.10	[0.51]	3.97	[0.62]
*B. Personal & household characteristics*						
Female (dummy, = 1 if yes)	0.55	[0.50]	0.55	[0.50]	0.55	[0.50]
Married (dummy, = 1 if yes)	0.92	[0.28]	0.93	[0.26]	0.91	[0.29]
Ethnic minority (dummy, = 1 if not ethnic Han)	0.01	[0.08]	0.01	[0.10]	0.004	[0.07]
Education (years)	6.59	[3.24]	6.74	[3.13]	6.47	[3.32]
Age (years)	43.75	[9.96]	43.76	[9.33]	43.74	[10.44]
Age squared	2013	[841.3]	2002	[798.9]	2023	[874.7]
Number of chronic conditions diagnosed	0.06	[0.25]	0.07	[0.29]	0.04	[0.21]
Working (dummy, = 1 if yes)	0.94	[0.23]	0.96	[0.19]	0.93	[0.26]
Number of children under age 6 in the household	1.94	[1.82]	1.76	[1.81]	2.08	[1.81]
Number of elders over age 55 in the household	2.11	[3.00]	1.82	[2.83]	2.35	[3.11]
Log of household income per capita (CPI-adjusted)	9.21	[1.33]	9.38	[0.91]	9.08	[1.58]
Number of observations (N)	2,155		969		1,186	

Notes: The numbers of observations reported (last row) are the largest numbers of observations in each of the panels. The number of observations used in the analysis is somewhat smaller due to missing information on certain variables.

### 2.2 Construction of diet-structure indicators

The CHNS recorded detailed information on sample individuals’ consumption of more than 1,500 food items during three consecutive days, allowing us to construct multiple indicators to measure their diet structure.

*Diet diversity score*. The first indicator, the diet-diversity score (DDS), counts the number of food groups an individual consumed over the past 24 h out of 12 recommended groups (Table 2, panel C). These recommended groups were chosen by experts at the Chinese Center for Disease Control and Prevention and the China Nutrition Association (CNA), with reference to the *Chinese Dietary Guidelines* of 2007 (CNA, 2008).^4^ If an individual's consumption of foods in a given group (e.g., “soybean and soybean products”) reaches or exceeds the minimum intake level recommended for that group (e.g., “25 g”), a score of one is assigned to that group for this individual; otherwise, a score of zero is assigned. By construction, the DDS ranges from zero to 12, with a higher score indicating a more diverse diet.^5^

**Table 2:** Food categories involved in the Diet Diversity Score and Diet Balance Index.

(1) Chinese Dietary Guidelines (CNA, 2008)	(2) Components in the Diet Balance Index (DBI)	(3) Food groups and corresponding intake thresholds involved in the Diet Diversity Score (DDS)
CDG-1: Eat a variety of foods, with cereals as the staple and a certain amount of coarse grains.	DBI-1: Diet variety (DDS1∼DDS12)	
	DBI-2: Grains	DDS-1: Rice and rice products (25 g)
		DDS-2: Wheat and wheat products (25 g)
		DDS-3: Corn, coarse grains, starchy roots, and their products (25 g)
CDG-2: Consume plenty of vegetables, fruits, and tubers.	DBI-3: Vegetables and fruits	DDS-4: Dark-colored vegetables (25 g)
		DDS-5: Light-colored vegetables (25 g)
		DDS-6: Fruits (25 g)
CDG-3: Consume milk, soybean, and their products every day.	DBI-4: Soybean and dairy products	DDS-7: Soybean and soybean products (5 g)
		DDS-8: Milk and dairy products (25 g)
CDG-4: Consume proper amounts of fish, poultry, eggs, and lean meat.	DBI-5: Animal protein	DDS-9: Red meat (livestock products) (25 g)
		DDS-10: Poultry and games (25 g)
		DDS-11: Egg (25 g)
		DDS-12: Aquatic products (25 g)
CDG-5: Reduce cooking oil; choose a light diet that is also low in salt.	DBI-6: Cooking oil, salt, and alcoholic beverages	
CDG-6: If you drink alcoholic beverages, do so in limited amounts.		
CDG-7: Avoid overeating and exercise every day to maintain healthy body weight.	n/a	
CDG-8: Rationally distribute the daily food intake among the three meals. If you take snacks, do so properly.	n/a	
CDG-9: Drink sufficient water every day; rationally choose beverages.	DBI-7: Drinking water	
CDG-10: Avoid unsanitary and spoiled foods.	n/a	

Source: He et al. (2009).

Notes: “Drinking water” is not available in the CHNS data.

While the DDS provides a quick and low-cost way to assess one's diet structure that can be adopted in many types of surveys, this indicator has a significant limitation: it does not take into account the actual *amount* of a food item one consumes beyond the recommended level. Thus, it lacks the power to detect one's over-eating (or under-eating) behavior, which could negatively impact one's health. The rich information in the CHNS data allows us to construct a more comprehensive indicator, the diet-balance index (DBI), to help capture one's over-consumption (and under-consumption) of essential foods.

*Diet-balance index*. The DBI was designed by experts at the Chinese Center for Disease Control and Prevention (He et al., 2009), based upon the principles of constructing the Healthy Eating Index and the Diet Quality Index, two indicators that are widely used in the literature (e.g., Kennedy et al., 1995; Patterson et al., 1994; Reedy et al., 2018). Adjusted to fit the Chinese context, the DBI contains seven components proposed in the *Chinese Dietary Guidelines* (CNA, 2008): diet variety, grains, vegetables and fruits, dairy and soybean products, animal products, condiments and alcoholic beverage, and drinking water (Table 2, panel B).^6^ Each component has one to four specific subcomponents, most of which overlap with the food categories involved in the DDS (Table 2, panel C). For example, the “vegetables” component (DBI-3) has two subcomponents (dark-colored vegetables and light-colored vegetables), and the “animal products” component (DBI-5) has four subcomponents (livestock, poultry, aquatic products, and eggs).

The construction of the DBI involves two steps. In the first step, a food group-specific score is assigned to an individual's consumption of a food group based on a set of scoring criteria. These criteria vary with recommended energy-intake levels set according to Chinese residents' gender, age, and intensity of daily physical activities. Table 3, for example, illustrates the criteria for an individual whose recommended level of energy intake is 2000 kcal/day; criteria for individuals with other recommended energy-intake levels are provided in He et al. (2009). The assigned score is positive if an individual's consumption of a food item exceeds the recommended level (“*over*-consumption”) and is negative (“*under-*consumption”) otherwise. In the second step, all step-one scores are summed up to yield the overall DBI. Based on this algorithm, an overall DBI score of *zero* indicatesa *balanced* diet; a positive (negative) score indicates over-consumption (under-consumption) of at least some foods.

**Table 3:** Scoring criteria for a male whose recommended energy intake is 2000 kcal/day in the definition of the Diet Balance Index.

Components	Sub-components	Score range	Score																
			-12	-10	-8	-6	-4	-3	-2	-1	0	1	2	3	4	6	8	10	12
DBI-1: Diet variety	Diet variety	-12–0	The score = 0 if a person consumes no less than 25 g (5 g for soybean and products) for a given food item included in the DDS; = -1 otherwise																
DBI-2: Grains	Grains	-12–12	<25	[25, 75)	[75, 125)	[125, 175)	[175, 225)		[225, 275)		[275, 325)		[325, 375)		[375, 425)	[425, 475)	[475, 525)	[525, 575)	≥575
DBI-3: Vegetables and fruits	Vegetables	-6–0				<1	[1, 175)		[175, 350)		≥350								
	Fruits	-6–0				<1	[1, 150)		[150, 300)		≥300								
DBI-4: Soybean and dairy products	Dairy	-6–0				[1,100)	[100, 200)		[200, 300)		≥300								
	Soybean	-6–0				<1	[1, 20)		[20, 40)		≥40								
DBI-5: Animal protein	Meat	-4–4					<1		[1, 25)		[25, 75)		[75, 125)		≥125				
	Fish	-4–0					<30	[30,45)	[45, 60)	[60, 75)	≥75								
	Eggs	-4–4					<1		[1, 25)		[35, 50)		[50,75)		≥75				
DBI-6: Cooking oil, salt, and alcoholic beverages	Cooking oil	0–4									≤25		(25,50]		>50				
	Salt	0–4									≤6		(6,12]		>12				
	Alcohol	0–4									≤25	(25,50]	(50,75]	(75,100]	>100				

Notes: The complete scoring system for all seven energy intake groups can be found in He et al. (2009).

Two advantages the DBI has over the DDS are worth noting. First, the DBI explicitly includes a “diet variety” component (DBI-1) that “mirrors” the DDS: this component assigns a score of negative one to a given food item involved in the DDS if a person consumes less than 25 g of that item (—5 g for soybean products), and a score of zero otherwise. As such, the DBI incorporates the DDS as one of its components while simultaneously capturing other aspects of one's diet structure. Second, the DBI involves several food items that are not considered in the DDS, such as cooking oil, condiments, and alcoholic beverages (DBI-6). Since the *Chinese Dietary Guidelines* do not encourage the consumption of these items, the DDS leaves these items out of its definition. By contrast, the DBI explicitly “punishes” excessive consumption of these items—as Table 3 shows, these items do not have negative scores, meaning that they could be over-consumed but would never be counted as being under-consumed by the DBI.

*DBI-distance*. It bears noting that the DBI also has a significant limitation. Over-consumption of certain foods and under-consumption of some other foods may jointly yield an overall DBI score that appears to be well-balanced. To address this problem, some researchers (e.g., Stookey et al., 2000) suggested summing up the absolute values of over-consumption (DBI-O) and under-consumption (DBI-U) scores to obtain a “DBI-distance” measure, i.e., DBI-distance = |DBI-O|+|DBI-U|, such that both over-consumption and under-consumption of foods would be penalized (by adding more points to the DBI-distance score). Yet, the DBI-distance measure is not flawless, either: it is insensitive to shocks that affect |DBI-O| and |DBI-U| in *opposite* directions. For example, if a policy simultaneously raises |DBI-O| (an undesirable effect) and reduces |DBI-U| (a desirable effect), the DBI-distance score may remain unchanged.

One way to avoid over-consumption and under-consumption of foods canceling out one another in the index is to compute two DBI sub-indicators that separately reflect over- (DBI-O) and under-consumption (DBI-U) of foods. Naturally, the DBI-O score sums all positive step-one DBI scores, and DBI-U is the sum of all negative step-one scores. The ranges of DBI-O/DBI-U scores reflect different levels of over/under-consumption: DBI-O scores within the ranges of 1–6, 7–13, 14–19, and 20–32 suggest, respectively, “fair”, “low”, “modest”, and “high” levels of over-consumption; DBI-U scores within the ranges of -1∼-12, -13∼-24, -25∼-36, and -37∼-60 suggest, respectively, “fair”, “low”, “modest”, and “high” levels of under-consumption (CNA, 2008).

**Figure 1:**
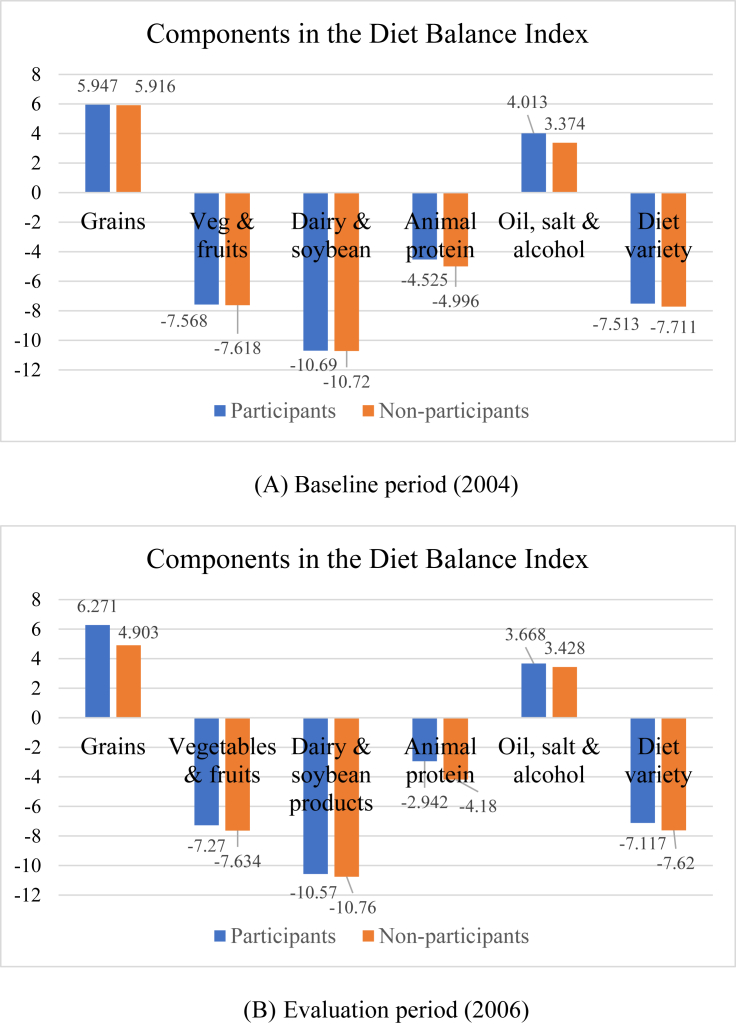
Mean Scores of Components in the Diet Balance Index at the Baseline. Notes: One of the 7 components in the DBI, “drinking water”, is unavailable in the CHNS data.

Table 1, panel A, reports summary statistics of the indicators discussed above for the analytical sample, measured at the baseline (2004). The overall DDS score (= 4.4) suggests that rural Chinese residents had a rather monotonous diet at the baseline, consuming only slightly more than four food categories out of the recommended 12. Consistent with this, the overall DBI score (= -21.1) suggests these individuals under-consume certain foods. Figure 1, displaying the DBI components measured at the baseline, reveals that sampled individuals not only under-consume certain foods (“vegetables and fruits”, ​“dairy and soybean products”, and ​“animal protein”), with a DBI-U of -34.2 (i.e., “modest” under-consumption), but also over-consume some other foods (“grains” ​and ​“cooking oil, condiments and alcoholic beverages”), with a DBI-O of 13.1 (i.e., “low” level of over-consumption). The main aim of this study, as previously noted, is to examine how enrollment in the NCMS affects these patterns.

## 3 Estimation method

### 3.1 The difference-in-differences framework

The sequential rollout of the NCMS across rural counties in China naturally suggests a difference-in-difference framework to evaluate its impacts, which has been adopted by Bai and Wu (2014), Chen and Jin (2012), and Wagstaff et al. (2009), among others, in their impact-evaluation studies. Formally, let a binary indicator *P* denote a sample individual's NCMS enrollment status: P=1 for participants and P=0 for nonparticipants. Following Lin and Lei (2009) and Yang and Wu (2015), we define individuals who had been enrolled in the program by 2006 as participants and those who had not as nonparticipants.^7^ Let another indicator, *T*, denote the time period of observation: T=0 for the pre-NCMS (baseline) period and T=1 for the post-implementation (evaluation) period. Although the NCMS was implemented in 2003, the CHNS data show that its coverage had reached only 3% in 2004, but went up to 40.6 % in 2006 and to 95% in 2009. Given the very small proportion of individuals covered in the NCMS in 2004, we set this year as the baseline and 2006 the evaluation period.

A first, crude estimator of the impact of NCMS enrollment is the difference in (the means of) a diet-structure outcome *Y* (e.g., DDS or DBI) of NCMS participants (P=1) between the baseline (T=0) and evaluation (T=1) periods:

(1) $$\delta^{P}=E\left(Y|P=1,T=1\right)-E\left(Y|P=1,T=0\right)$$

While the estimator *δ*^*P*^ does capture the program's impact, it may also capture the influence of other factors such as local economic conditions and food availability. These factors may change over time, causing rural Chinese residents' diet quality to improve between the two periods *even in the absence of the program*. In that case, *δ*^*P*^ will be a biased estimator of the program's impact.

Under the “parallel-trend” assumption that these “other factors” have a similar impact on both participants and nonparticipants (—such that the time trends of *E*(*Y*) in the two groups are similar *in the absence of the program*), one can refine the estimator *δ*^*P*^ by subtracting from it the change in the mean of *Y* among *nonparticipants* between the two periods, $\delta^{NP}=E\left(Y|P=0,T=1\right)-E\left(Y|P=0,T=0\right)$, which serves as the counterfactual for the change in the mean of *Y* among participants between the two periods. This refinement leads to a difference-in-difference estimator:

(2) $$\delta^{DID}=\delta^{P}-\delta^{NP}=\left[E\left(Y|P=1,T=1\right)-E\left(Y|P=1,T=0\right)\right]-\left[E\left(Y|P=0,T=1\right)-E\left(Y|P=0,T=0\right)\right]$$

It is well-established that the parameter $\delta^{DID}$ can be estimated from the following model:

(3) $$Y_{it}=\beta_{0}+\beta_{P}P_{i}+\beta_{T}T+\delta^{DID}\left(P_{i}\times T\right)+\varepsilon_{it}$$

where the subscript *i* refers to individuals and *t* denotes time periods. A set of observed covariates $X_{it}$ (including exogenous personal, household, and community characteristics) can be added to the model to help assess the robustness of estimation results and improve estimation precision:

(4) $$Y_{it}=\beta_{0}+\beta_{P}P_{i}+\beta_{T}T+\delta^{DID}\left(P_{i}\times T\right)+X_{it}\beta_{X}+\varepsilon_{it}$$

### 3.2 Combining DID estimation with propensity score matching

Another way to control for observed characteristics is to perform propensity score matching (PSM) using the characteristics observed at the baseline ($X_{i0}$) and then perform DID estimation using the matched sample over the “common support” region.^8^ There is more than one way to implement the DID-PSM technique. We follow Galiani et al. (2005) and perform two types of DID-PSM estimation. The first is to estimate Eq. (3) only with observations over the common support (*C*) region:

(5) $$Y_{it}=\beta_{0}+\beta_{P}P_{i}+\beta_{T}T+\delta^{DID}\left(P_{i}\times T\right)+\varepsilon_{it}|\hat{P_{i}}\left(X_{i0}\right)\in C$$

where $\hat{P_{i}}\left(X_{i0}\right)$ is the estimated propensity scores, i.e., the estimated probabilities of NCMS participation by probit or logit using $X_{i0}$ as predictors. This approach, also recommended by Khandker et al. (2011), is similar to an interval matching but with a somewhat different weighting scheme.

The second approach is to calculate the difference in outcome *Y* between the evaluation and baseline periods for each individual *i*, $\text{Δ}Y_{i}=Y_{i,2006}-Y_{i,2004}$, take the difference of Δ*Y* between matched pairs, and then average over all matched pairs to yield the DID-PSM estimate (Glewwe and Todd, 2022). In the analysis, we use Kernel matching to find matches for participants. Accordingly, the DID-PSM estimator is constructed as:

(6) $$\delta^{DID-PSM}=\frac{1}{N^{P}}\left[\underset{i\varepsilon \left\{P=1\right\}}{\sum }\text{Δ}Y_{i}^{P}-\underset{j\varepsilon \left\{P=0\right\}}{\sum }\omega \left(i,j\right)\text{Δ}Y_{j}^{NP}\right]$$

where the Kernel weights ω(i,j) are defined as:

(7) $$\omega \left(i,j\right)=\frac{K\left(\frac{{\hat{P}}_{j}-{\hat{P}}_{i}}{B_{n}}\right)}{\sum_{i\varepsilon C}K\left(\frac{{\hat{P}}_{j}-{\hat{P}}_{i}}{B_{n}}\right)}$$

where *K*(·) is a Kernel function and *B*_*n*_ is a bandwidth parameter. In the analysis reported below, the kernel weights ω(i,j) were obtained by executing the Stata command “psmatch2” with the “kernel” option, which performs local linear regression matching with an Epanechnikov kernel.

We opt to use Kernel matching because other algorithms, such as nearest-neighbor matching, caliber matching, and interval matching, may encounter the problem that only a small subset of nonparticipants fall within the common support. Kernel matching circumvents this problem by using a weighted average of all nonparticipants to construct the “counterfactual” match for each participant.

While both approaches are used in our analysis, the first approach is preferred in our setting because the second does not allow for the inclusion of community fixed effects (FEs) in the estimation. Note that many individuals (*N* = 497) resided in counties where the NCMS was not available in 2006. For these individuals, the identity of their residing communities (captured by community FEs) perfectly predicts their NCMS enrollment status, pushing them out of the common support region. As such, the inclusion of community FEs would substantially reduce the common support region and (thus) the size of the analytical sample. In contrast, the first approach, performing DID over the common support region constructed without community FEs, allows us to include community FEs in the second step. Simply put, the first approach enables us to exploit more information in estimation.^9^

### 3.3 Testing the parallel-trend assumption

Note that the validity of DID estimates (even when combined with PSM) hinges on the plausibility of the “parallel-trend” assumption. With the availability of another wave of data collected prior to NCMS implementation, one can perform a falsification test using the two pre-NCMS datasets. Let $T_{0}=0$ denote the earlier period (2000 in our setting) and $T_{0}=1$ denote the later, “placebo” evaluation period (2004 in our case). Since no one was enrolled in the NCMS in these two pre-NCMS periods, the time trend of *Y* for either group (participants or nonparticipants) should not pick up any impact of NCMS enrollment. Thus, if the parallel-trend assumption is plausible, one would expect the pre-NCMS time trend of *Y* for the participants, $\left[E\left(Y|P=1,T_{0}=1\right)-E\left(Y|P=1,T_{0}=0\right)\right]$, and that for the nonparticipants, $\left[E\left(Y|P=0,T_{0}=1\right)-E\left(Y|P=0,T_{0}=0\right)\right]$, to be similar.

Formally, the “parallel-trend” assumption implies that

(8) $$\delta_{0}^{DID}=\left[E\left(Y|P=1,T_{0}=1\right)-E\left(Y|P=1,T_{0}=0\right)\right]-\left[E\left(Y|P=0,T_{0}=1\right)-E\left(Y|P=0,T_{0}=0\right)\right]=0.$$

Thus, one would expect a DID model applied to the two pre-NCMS waves of data,

(9) $$Y_{it}=\alpha_{0}+\alpha_{P}P_{i}+\alpha_{T}T_{0}+\delta_{0}^{DID}\left(P_{i}\times T_{0}\right)+\varepsilon_{it},$$

to yield a small and statistically insignificant estimate of $\delta_{0}^{DID}$. Again, a set of covariates $X_{it}$ can be added in the model (Eq. (9)) as control variables or used as predictors of propensity scores $\hat{P_{i}}\left(X_{i0}\right)$.

## 4 Results

### 4.1 Impacts of NCMS enrollment on diet structure

**Figure 2:**
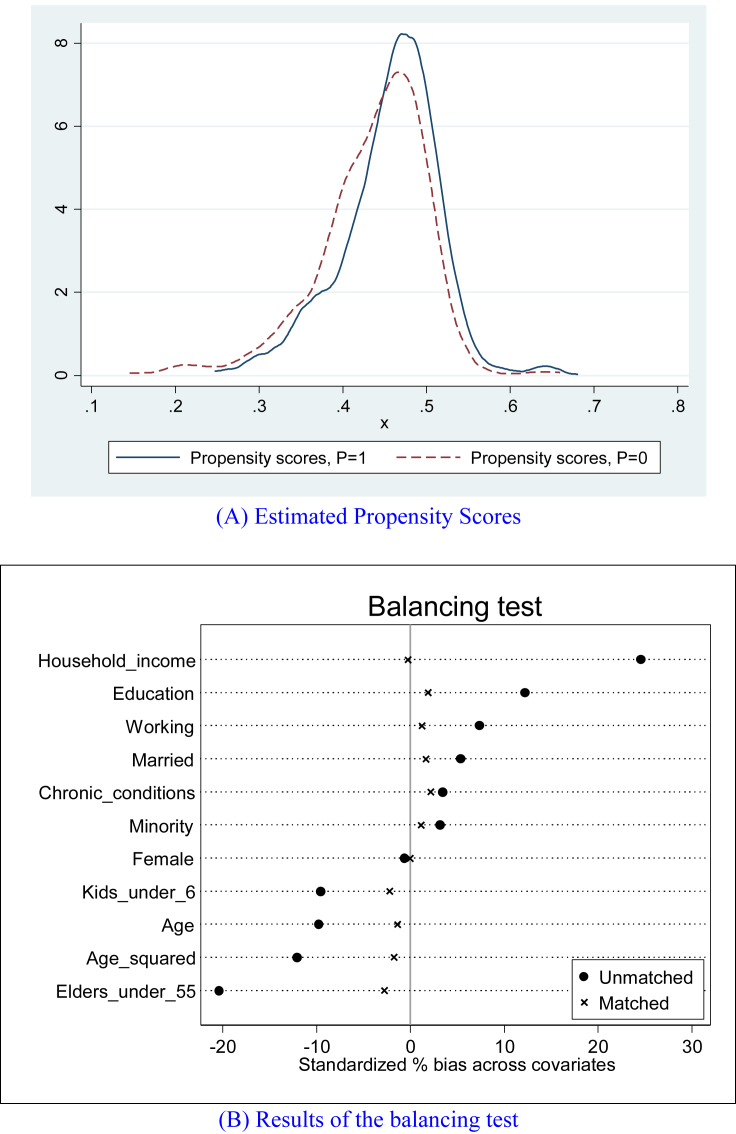
Results of propensity scores estimation and the balancing test. Note: the common support region is the overlapping region between participants (*P* = 1) and nonparticipants' (*P* = 0) estimated propensity scores.

Table 4 reports the main findings of this study: DID estimates of the impacts of NCMS enrollment on rural Chinese individuals’ diet structure, as measured by the diet-diversity score (DDS) (panel A) and the diet-balance index (DBI) (panel B). Four specifications are adopted for each outcome: the first is the simplest DID model, estimating Eq. (3) with no other covariates; the second adds a set of personal and household characteristics, and the full set of community FEs, estimating Eq. (4); the third model estimates Eq. (5), i.e., the same specification as the second model (Eq. (3)), but with only observations over the common support (Figure 2, panel A); the last, a DID-PSM model, estimates Eq. (6) using only observations paired by Kernel matching (—results of the first-stage propensity score estimation and the balancing test are reported in Table 5 and Figure 2, panel B, respectively).^10^

**Table 4:** Difference-in-differences estimates of the impacts of NCMS enrollment on rural Chinese residents’ diet structure.

	(1)	(2)	(3)	(4)	(5)	(6)	(7)	(8)
Outcome:	A. Diet-diversity score (DDS)				B. Diet-balance index (DBI)			
Estimator:	DID	DID	DID (on common Support)	DID-PSM (Kernel matching)	DID	DID	DID (on common Support)	DID-PSM (Kernel matching)
Treatment effects (*P*×*T*)	0.305∗∗	0.343∗∗	0.344∗∗	0.331∗∗∗	2.577∗∗	2.628∗∗	2.656∗∗	2.451∗∗∗
	(0.145)	(0.147)	(0.144)	(0.104)	(1.003)	(1.030)	(1.029)	(0.903)
Participants (*P*)	0.198	-0.096	-0.108		1.360	-0.797	-0.762	
	(0.139)	(0.101)	(0.104)		(1.057)	(0.685)	(0.740)	
Post-NCMS (*T*)	0.091	0.054	0.078		-0.133	-0.289	-0.077	
	(0.112)	(0.113)	(0.107)		(0.798)	(0.812)	(0.808)	
Female (=1 if yes)		0.019	0.016			2.340∗∗∗	2.285∗∗∗	
		(0.020)	(0.022)			(0.214)	(0.224)	
Married (=1 if yes)		0.107∗	0.094			1.051∗∗	1.060∗∗	
		(0.060)	(0.058)			(0.431)	(0.465)	
Ethnic minority (=1 if not ethnic Han)		0.042	-0.151			-2.477∗∗	-2.350	
		(0.191)	(0.207)			(1.208)	(1.591)	
Education (years)		0.023∗∗∗	0.024∗∗∗			0.124∗∗∗	0.124∗∗∗	
		(0.006)	(0.006)			(0.040)	(0.040)	
Age (years)		-0.021	-0.016			-0.024	-0.031	
		(0.014)	(0.014)			(0.090)	(0.094)	
Age squared		0.000	0.000			0.000	0.000	
		(0.000)	(0.000)			(0.001)	(0.001)	
Number of chronic conditions		-0.031	-0.033			0.190	0.202	
		(0.049)	(0.049)			(0.413)	(0.410)	
Working (=1 if yes)		0.004	-0.015			-0.155	-0.271	
		(0.064)	(0.065)			(0.487)	(0.534)	
Number of kids under 6		0.004	-0.001			-0.048	-0.049	
		(0.011)	(0.011)			(0.080)	(0.086)	
Number of elders over 55		0.009	0.008			-0.089∗	-0.093∗∗	
		(0.006)	(0.006)			(0.047)	(0.047)	
Income per capita (yuan, log)		0.054∗∗∗	0.061∗∗∗			0.243∗∗	0.233∗∗	
		(0.018)	(0.019)			(0.106)	(0.106)	
Community FE	No	Yes	Yes	No	No	Yes	Yes	No
N	4,301	4,223	3,796	1,876	4,240	4,164	3,740	1,876
R^2^	0.043	0.369	0.372		0.038	0.394	0.405	

Notes: Robust standard errors in parentheses, adjusted at the community level. There are 874 participants and 1,002 matched nonparticipants in the DID-PSM (columns 4 and 8) analyses. Standard errors for kernel matching (local linear regression matching with an Epanechnikov kernel) estimates are bootstrapped using 100 replications. ∗∗∗p < 0.01, ∗∗p < 0.05, ∗p < 0.10.

**Table 5:** Results of propensity scores estimation (probit estimates).

Outcome variable: NCMS participation in 2004	(1)	(2)
Variables	Coefficients	Standard errors
Female (dummy, = 1 if yes)	0.017	(0.060)
Married (dummy, = 1 if yes)	-0.083	(0.109)
Ethnic minority (dummy, = 1 if not ethnic Han)	0.511	(0.360)
Education (years)	0.012	(0.010)
Age (years)	0.111∗∗∗	(0.025)
Age squared	-0.001∗∗∗	(0.000)
Number of chronic conditions	0.441∗∗∗	(0.117)
Working (=1 if yes)	0.356∗∗∗	(0.131)
Number of kids under 6	-0.073∗∗∗	(0.016)
Number of elders over 55	-0.025∗∗	(0.010)
Income per capita (yuan, log)	0.131∗∗∗	(0.025)
Constant	-3.666∗∗∗	(0.555)
N	2,112	
Pseudo R^2^	0.0374	

Notes: ∗∗∗p < 0.01, ∗∗p < 0.05, ∗p < 0.1.

The results in Table 4 tell a consistent story: enrollment in the NCMS significantly improved rural Chinese residents' diet structure, measured by either the DDS (columns 1–4) or the DBI (columns 5–8). The increase in the DDS (by 0.31–0.34) suggests that, as a result of NCMS enrollment, rural Chinese residents consumed about 0.3 more food groups out of the 12 groups recommended by the *Chinese Dietary Guidelines* (CNA, 2008). Given the “control” mean of the DDS at the baseline, 4.3 (Table 1, panel B), this effect is equivalent to a 7–8% increase in rural residents' diet diversity. The increase in the DBI (by 2.5–2.7 points), together with the negative baseline “control” mean of -34.4 (Table 1, panel B), suggests that, overall, NCMS enrollment reduced the incidence of under-consumption of foods among the insured. Echoing the increases in the DDS and the DBI, panels A–D of Table 6 show that NCMS enrollment significantly raised the insured's nutrition intake levels. More specifically, NCMS enrollment increases rural residents' carbohydrate intake by 11.3%, protein intake by 8.6–9.3%, and fat intake by 6.6–7.3% (although imprecisely estimated), leading to an increase in their total calorie intake by 8.5–9.2%.

**Table 6:** Difference-in-differences estimates of impacts of NCMS enrollment on rural Chinese residents’ diet structure and nutrition intakes.

	(1)	(2)	(3)	(4)
Outcome:	Nutrition intakes		Diet structure	
Estimator:	DID with controls; over the common support	DID-PSM (Kernel matching)	DID with controls; over the common support	DID-PSM (Kernel matching)
	A. log(calorie)		E. Overall DBI	
Estimate of NCMS-enrollment effect (${\hat{\delta }}^{DID}$)	0.093∗∗	0.087∗∗∗	2.656∗∗	2.451∗∗∗
	(0.036)	(0.031)	(1.029)	(0.903)
N	3,828	1,876	3,740	1,876
R^2^	0.349		0.405	
	B. log(carbohydrate)		F. DBI-O (over-consumption)	
Estimate of NCMS-enrollment effect (${\hat{\delta }}^{DID}$)	0.113∗∗∗	0.112∗∗∗	1.097∗	0.945∗
	(0.040)	(0.023)	(0.585)	(0.525)
N	3,823	1,876	3,740	1,876
R^2^	0.388		0.383	
	C. log(protein)		G. DBI-U (under-consumption)	
Estimate of NCMS-enrollment effect (${\hat{\delta }}^{DID}$)	0.073∗	0.071∗∗	1.539∗∗	1.519∗∗∗
	(0.042)	(0.030)	(0.601)	(0.550)
N	3,825	1,876	3,761	1,876
R^2^	0.256		0.358	
	D. log(fat)		H. DBI distance (=|DBI-O|+|DBI-U|)	
Estimate of NCMS-enrollment effect (${\hat{\delta }}^{DID}$)	0.095	0.078	0.036	-0.029
	(0.088)	(0.078)	(0.562)	(0.558)
N	3,825	1,876	3,761	1,876
R^2^	0.300		0.299	

Notes: Robust standard errors in parentheses, adjusted for clustering at the community level. There are 874 participants and 1,002 matched nonparticipants in the DID-PSM (columns 4 and 8) analyses. Standard errors for kernel matching estimates are bootstrapped using 100 replications.

∗∗∗p < 0.01, ∗∗p < 0.05, ∗p < 0. 10.

Yet more is not always better. The above results—increases in diet diversity, overall diet balance, and nutrition intakes—might have been accompanied by *over-consumption* of certain foods. As discussed above, over-consumption of some foods and under-consumption of some other foods may jointly yield an overall DBI score that suggests a well-balanced diet structure. The DDS and the nutrition intake-based indicators also lack the power to detect potential over-consumption behavior. To check the possibility of over-consumption of foods, Panels F and G of Table 6 examine the two DBI sub-indicators separately, revealing an informative pattern that the overall DBI, the DDS, and the nutrition-intake indicators all failed to discover.^11^ NCMS enrollment drives up the overall DBI by *raising* the incidence of over-consumption of some foods (—DBI-O scores *increased*, becoming more positive) while *reducing* that of under-consumption of (some other) foods (—DBI-U scores also *increased*, becoming less negative). The DBI-distance measure picks up neither effect because the increase in the absolute value of DBI-O (—more positive, so |DBI-O| becomes larger) and the decrease in that of DBI-U (—less negative, so |DBI-U| becomes smaller) essentially cancel one another out.

### 4.2 Robustness check

**Figure 3:**
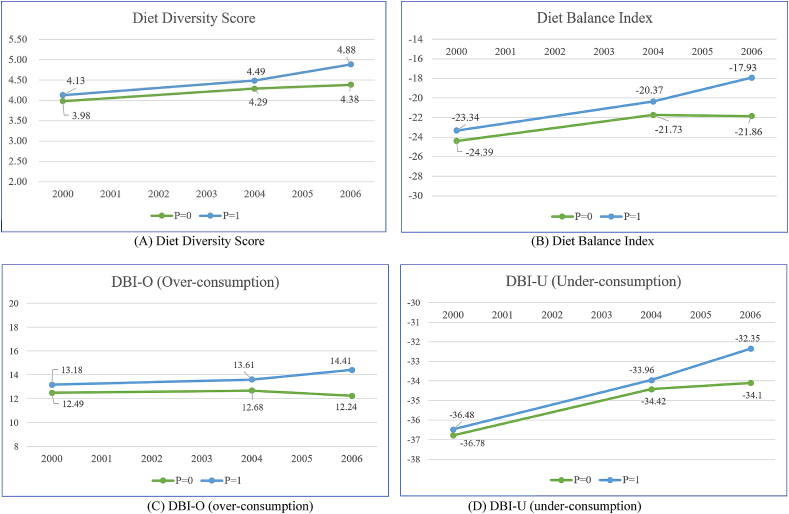
Time trends in diet structure (2000–2006).

The validity of the above results (i.e., impacts of NCMS enrollment on the DDS, overall DBI, and two DBI sub-indicators)^12^ hinges on that of our DID design. Thus, we performed a series of checks to assess the validity of these results. The first check concerns the plausibility of the parallel-trend assumption (8). We test this assumption using the two pre-NCMS datasets ($T_{0}=0$ for 2000 and $T_{0}=1$ for 2004) to estimate Eq. (9). The results, reported in Table 7, reveal no sign of violation of the parallel-trend assumption. Regardless of the empirical specifications adopted, none of the estimates of $\delta_{0}^{DID}$ in Eq. (9) is statistically significant for any diet-structure indicators discussed above. Figure 3 visualizes these results, clearly showing that the time trends of these indicators for participants and nonparticipants were indeed parallel in the pre-NCMS period (2000–2004), despite their divergence in the post-implementation period (2004–2006).

**Table 7:** Results of testing the parallel-trend assumption.

Outcome variables	(1)	(2)	(3)	(4)	(5)	(6)	(7)	(8)
	A. Diet diversity score (DDS)				B. Diet balance index (DBI)			
	DID	DID	DID on common support	DID-PSM (Kernel matching)	DID	DID	DID on common support	DID-PSM (Kernel matching)
Placebo effect: *P×T*_0_ ($\delta_{0}^{DID}$)	0.045	0.017	-0.022	-0.003	0.316	0.627	0.476	0.448
	(0.142)	(0.143)	(0.147)	(0.104)	(1.259)	(1.221)	(1.279)	(1.098)
*P*	0.152	0.100	0.119		1.044	-0.119	0.082	
	(0.126)	(0.121)	(0.124)		(1.019)	(0.919)	(0.979)	
*T*_0_	0.313∗∗∗	0.317∗∗∗	0.325∗∗∗		2.655∗∗	2.502∗∗	2.698∗∗	
	(0.114)	(0.115)	(0.121)		(1.042)	(1.028)	(1.091)	
Controls	No	Yes	Yes	No	No	Yes	Yes	No
Community FE	No	Yes	Yes	No	No	Yes	Yes	No
N	3,916	3,765	3,445	1,876	3,758	3,614	3,301	1,876
R^2^	0.031	0.290	0.271		0.034	0.312	0.317	
	C. DBI-O: Over-consumption				D. DBI-U: Under-consumption			
Placebo effect: *P×T*_0_ ($\delta_{0}^{DID}$)	0.239	0.407	0.446	0.386	0.151	0.082	-0.097	-0.003
	(0.771)	(0.773)	(0.796)	(0.652)	(0.616)	(0.610)	(0.629)	(0.571)
*P*	0.689	-0.506	-0.479		0.308	0.519	0.689	
	(0.520)	(0.580)	(0.615)		(0.592)	(0.519)	(0.541)	
*T*_0_	0.194	0.210	0.283		2.365∗∗∗	2.400∗∗∗	2.509∗∗∗	
	(0.608)	(0.635)	(0.661)		(0.498)	(0.499)	(0.520)	
Controls	No	Yes	Yes	No	No	Yes	Yes	No
Community FE	No	Yes	Yes	No	No	Yes	Yes	No
N	3,758	3,614	3,301	1,876	3,838	3,692	3,375	1,876
R^2^	0.008	0.243	0.251		0.065	0.342	0.341	

Notes: Robust standard errors in parentheses, adjusted for clustering at the community level. There are 874 participants and 1,002 matched nonparticipants in the DID-PSM (columns 4 and 8) analyses. Standard errors for kernel matching (local linear regression matching with an Epanechnikov kernel) estimates are bootstrapped using 100 replications.

∗∗∗p < 0.01, ∗∗p < 0.05, ∗p < 0.1.

Another concern is that the effect of NCMS enrollment on food consumption may not be immediate,^13^ and thus the DID estimates discussed in section 4.1 might have captured something else. It is, therefore, worth examining how the time duration between NCMS enrollment and the CHNS interview may impact our estimation results. Although the CHNS did not collect information on the exact timing of one's NCMS enrollment, it did record each respondent's date of interview. We can still gain a sense of how the duration from NCMS enrollment affects our estimates by adding the interview date as an additional control. This is because no matter when a respondent was enrolled, the duration from NCMS enrollment will be longer if this individual was interviewed at a later date. As such, the inclusion of interview date (defined as the number of days from January 1, 2006) would capture at least some effect of the time from NCMS enrollment. If most individuals from a given community enrolled in the program at around the same time, controlling for community fixed effects would help capture the full effect. The results (not reported here but made available upon request) show that conditional on community fixed effects, interview date has little explanatory power for sample individuals' diet structure, greatly alleviating the concern about the time-from-enrollment effect. Meanwhile, the DID estimates of NCMS-enrollment effects remain very similar to those reported in Tables 4 and 6.

The third concern is that even if the parallel-trend assumption holds, the above DID framework lacks the power to control for unobserved factors that vary between the baseline and evaluation periods. To address this concern, we follow Lei and Lin (2009) and Green et al. (2021) and use the availability of the NCMS in one's residing community in the evaluation period as an instrumental variable (IV) for his/her NCMS enrollment decision as an alternative modeling strategy. Under the assumption that the availability of the NCMS was exogenous to rural residents' food consumption decisions and only affected their food consumption through NCMS enrollment, this IV can provide consistent estimates of the impacts of NCMS enrollment. Based on data from the 2006 wave, IV estimates of the impacts of NCMS enrollment on the DDS, overall DBI, two DBI sub-indicators, and DBI-distance (Table 8) are all comparable to their DID counterparts reported in Tables 4 and 6.

**Table 8:** Instrumental variable estimates of impacts of NCMS enrollment.

Variables	(1)	(2)	(3)	(4)	(5)	(6)
	DDS	DBI	DBI-O	BDI-U	DBI-Distance	NCMS enrollment
						First-stage regression
Availability of NCMS in the village						0.795∗∗∗
						(0.030)
NCMS enrollment (P)	0.395∗∗	3.148∗∗	1.685∗	1.455∗	-0.356	
	(0.179)	(1.495)	(0.866)	(0.800)	(0.680)	
Female	0.074∗∗	2.241∗∗∗	0.918∗∗∗	1.326∗∗∗	-0.356∗∗	-0.001
	(0.032)	(0.305)	(0.212)	(0.151)	(0.173)	(0.009)
Married	0.022	1.211	0.891∗∗	0.325	0.490	0.005
	(0.096)	(0.753)	(0.440)	(0.439)	(0.416)	(0.024)
Education (years)	0.045∗∗∗	0.113	-0.015	0.129∗∗∗	-0.151∗∗∗	-0.000
	(0.008)	(0.069)	(0.040)	(0.038)	(0.036)	(0.002)
Age (years)	-0.001	-0.040	0.020	-0.059	0.049	0.011
	(0.022)	(0.193)	(0.123)	(0.101)	(0.113)	(0.007)
Age squared	0.000	0.001	-0.000	0.001	-0.001	-0.000∗
	(0.000)	(0.002)	(0.001)	(0.001)	(0.001)	(0.000)
Number of chronic conditions	-0.001	0.484	0.338	0.148	0.062	0.022
	(0.067)	(0.513)	(0.361)	(0.283)	(0.385)	(0.023)
Working (=1 if yes)	-0.130	-0.404	0.144	-0.551	0.359	-0.007
	(0.106)	(0.671)	(0.363)	(0.411)	(0.428)	(0.021)
Number of kids under 6	-0.016	-0.200	-0.148	-0.052	0.014	0.000
	(0.016)	(0.145)	(0.093)	(0.072)	(0.069)	(0.006)
Number of elders over 55	0.009	-0.113	-0.094∗	-0.021	0.004	0.004
	(0.009)	(0.091)	(0.055)	(0.046)	(0.044)	(0.003)
Income per capita (yuan, log)	0.072∗∗∗	0.369∗∗	0.061	0.308∗∗∗	-0.358∗∗∗	-0.001
	(0.020)	(0.169)	(0.100)	(0.108)	(0.117)	(0.007)
Constant	3.503∗∗∗	-28.467∗∗∗	8.960∗∗∗	-37.449∗∗∗	45.828∗∗∗	-0.191
	(0.531)	(4.482)	(2.879)	(2.472)	(2.863)	(0.162)
Province fixed effects	Yes	Yes	Yes	Yes	Yes	Yes
N	2,161	2,132	2,132	2,136	2,136	2,161
R^2^	0.306	0.236	0.228	0.224	0.208	0.630

Notes: Only observations in the 2000 wave are used in the estimation. Robust standard errors in parentheses, adjusted for clustering at the community level. F-test of the significance of the IV in the first-stage regression is 695.9044, much larger than the rule-of-thumb value of 10.

∗∗∗p < 0.01, ∗∗p < 0.05, ∗p < 0.1.

### 4.3 Impacts of NCMS enrollment on the consumption of specific food groups

To help understand the above findings on the DDS and DBI more deeply, Table 9 further explores the effects of NCMS enrollment on the insured's consumption of specific food items/groups involved in these indicators. Columns (2) and (4) reveal that NCMS enrollment has significantly raised the insured's consumption of three food groups, i.e., rice, fruits, and livestock products, among the 12 recommended groups in the DDS. Reporting estimated effects on the DBI's components, column (6) reveals more informative patterns than those seen in columns (2) and (4). While fruits and livestock products were originally under-consumed, rice was originally over-consumed (Table 9, column 5; Figure 1, panel A). As such, unlike the desirable impacts of NCMS enrollment on fruits and livestock consumption, its stimulation effect on rice consumption may be health-threatening.

**Table 9:** Difference-in-differences estimates of impacts of NCMS coverage on the consumption of specific food groups.

Estimators	(1)	(2)	(3)	(4)	(5)	(6)
	A. Amount consumed		B. Food groups in DDS		C. Components of DBI	
	Participants mean at baseline (g)	DID on common support	Participants mean at baseline (g)	DID on common support	Participants mean at baseline (g)	DID on common support
*Diet diversity*					-7.513	0.343∗∗∗
					[0.965]	(0.077)
*Grains*						
Rice	283.950	33.208∗∗	0.788	0.040∗∗	5.947 [5.045]	1.190∗∗∗ (0.345)
	[211.684]	(13.564)	[0.366]	(0.018)		
Wheat	181.555	-3.528	0.619	0.011		
	[185.147]	(15.014)	[0.399]	(0.031)		
Other grains	106.501	6.225	0.505	0.033		
	[126.327]	(11.814)	[0.394]	(0.044)		
*Vegetables & fruits*						
Dark vegetables	50.988	10.572	0.222	-0.020	-7.568 [1.514]	0.385∗∗∗ (0.134)
	[96.253]	(9.164)	[0.324]	(0.035)		
Light vegetables	342.228	-3.748	0.896	0.019		
	[213.750]	(21.167)	[0.217]	(0.025)		
Fruits	9.361	28.045∗	0.043	0.066∗∗		
	[47.589]	(14.758)	[0.178]	(0.031)		
*Dairy & bean products*						
Dairy products	3.442	-1.931	0.013	-0.007∗	-10.691 [1.691]	0.153 (0.137)
	[30.855]	(1.261)	[0.104]	(0.004)		
Soybean products	38.195	2.262	0.208	0.028		
	[64.690]	(6.127)	[0.272]	(0.033)		
*Animal protein*						
Livestock	220.222	76.925∗∗∗	0.679	0.135∗∗	-4.525 [3.753]	1.012∗∗∗ (0.262)
	[212.350]	(28.708)	[0.410]	(0.052)		
Poultry	26.776	-2.722	0.174	0.005		
	[61.475]	(5.272)	[0.328]	(0.034)		
Aquatic products	17.560	2.747	0.084	0.012		
	[50.603]	(4.183)	[0.176]	(0.018)		
Egg	23.697	2.500	0.258	0.021		
	[40.070]	(3.658)	[0.291]	(0.037)		
*Oil, condiments & alcoholic beverages*						
Cooking oil	43.915	-10.001			4.013 [2.327]	-0.493∗∗∗ (0.184)
	[34.547]	(6.472)				
Salt	10.678	-2.703				
	[11.627]	(5.445)				
Alcohol	8.531	6.464				
	[55.227]	(5.242)				
N	954	3,790	954	3,796	954	3,755

Notes: Columns (1), (3) and (5) report the means of food groups of interest consumed in the participants group observed at the baseline. Each cell in columns (2), (4) and (6) presents an DID estimate of $\delta^{DID}$ in a regression performed on the common support.

Standard deviations in brackets; robust standard errors in parentheses, adjusted for clustering at the community level.

∗∗∗p < 0.01, ∗∗p < 0.05, ∗p < 0.10.

Note also that the DBI detects effects that are not captured by the DDS or the nutrition-based indicators. In particular, column (6) of Table 9 reveals that NCMS enrollment reduces the insured's consumption of “cooking oil, condiments, and alcoholic beverages (DBI-6)”, which is not part of the DDS or the nutrition-based indicators. This finding, again, demonstrates that the DBI outperforms other indicators in assessing the effect of NCMS enrollment on the insured's diet structure.

### 4.4 Heterogeneity

Another way to understand the effects of NCMS enrollment is to examine potential heterogeneity in the effects across different subgroups of individuals. As suggested in columns (2), (3), (6), and (7) of Table 4, female, married, better-educated, and relatively wealthier individuals tend to have a more diverse and balanced diet while those living with elders tend to have a less balanced diet. It is thus natural to explore potential heterogeneity in NCMS-enrollment effects across subgroups defined by different values of these factors. Specifically, we separate the analytical sample by the median values of these variables and re-estimate the DID specifications of columns (3) and (7) in Table 4.

Table 10 reports the results. Interestingly, male, less-educated, and unmarried individuals, whose diet structure was relatively less balanced (Table 4, panel B), caught up with female, more-educated, and married individuals in diet balance as a result of NCMS enrollment. Note that while the incidences of over-consumption and under-consumption of foods both increased due to NCMS enrollment, the increase in the latter outweighs that in the former. This finding suggests that PHI programs may reduce inequality in diet diversity and balance among the insured. However, family income works in the opposite direction, as NCMS enrollment raised scores of all diet-structure indicators for relatively wealthy individuals whose diet was already more diverse and balanced than their less-wealthy counterparts (Table 4, columns 2–3, 6–7).

**Table 10:** Heterogeneity in the effects of NCMS enrollment (DID estimates over the common support).

Outcome variables	(1)	(2)	(3)	(4)	(5)	(6)	(7)	(8)
	DDS	DBI	DBI-O	DBI-U	DDS	DBI	DBI-O	DBI-U
	A. Female				B. Male			
Estimates of NCMS-enrollment effects	0.333∗∗	2.235∗∗	0.793	1.429∗∗	0.362∗∗	3.210∗∗∗	1.484∗∗	1.700∗∗
	(0.143)	(1.057)	(0.626)	(0.606)	(0.163)	(1.130)	(0.649)	(0.663)
N	2,069	2,041	2,041	2,051	1,712	1,684	1,684	1,695
R^2^	0.374	0.406	0.385	0.364	0.392	0.426	0.411	0.373

	C. Not married				D. Married			
Estimates of NCMS-enrollment effects	0.814∗∗	4.916∗	1.642	2.887∗	0.308∗∗	2.456∗∗	1.010∗	1.444∗∗
	(0.341)	(2.682)	(1.564)	(1.617)	(0.145)	(1.026)	(0.590)	(0.598)
N	271	263	263	269	3,510	3,462	3,462	3,477
R^2^	0.511	0.555	0.557	0.532	0.379	0.413	0.389	0.364

	E. Education < median (6 years)				F. Education ≥ median (6 years)			
Estimates of NCMS-enrollment effects	0.454∗∗∗	3.257∗∗∗	1.293∗∗	1.947∗∗∗	0.259	2.124∗	0.877	1.235∗
	(0.153)	(1.120)	(0.644)	(0.663)	(0.166)	(1.229)	(0.738)	(0.678)
N	1,997	1,965	1,965	1,977	1,784	1,760	1,760	1,769
R^2^	0.404	0.432	0.382	0.401	0.375	0.417	0.421	0.357
	G. Household income per capita ≤ median				H. Household income per capita > median			
Estimates of NCMS-enrollment effects	0.224	1.608	0.533	1.080	0.480∗∗	3.880∗∗∗	1.739∗∗∗	2.110∗∗∗
	(0.163)	(1.217)	(0.697)	(0.687)	(0.197)	(1.142)	(0.645)	(0.779)
N	1,892	1,861	1,861	1,874	1,889	1,864	1,864	1,872
R^2^	0.394	0.414	0.389	0.396	0.388	0.459	0.446	0.362
	I. Number of elders over 55 = 0				J. Number of elders over 55 ≥ 1			
Estimates of NCMS-enrollment effects	0.312∗	2.895∗∗	1.168∗	1.682∗∗	0.372∗∗	2.358∗	1.026	1.334∗
	(0.171)	(1.221)	(0.688)	(0.731)	(0.163)	(1.207)	(0.715)	(0.692)
N	1,943	1,911	1,911	1,922	1,838	1,814	1,814	1,824
R^2^	0.399	0.416	0.412	0.367	0.399	0.450	0.414	0.405

Notes: Robust standard errors in parentheses, adjusted for clustering at the community level.

∗∗∗p < 0.01, ∗∗p < 0.05, ∗p < 0.1.

## 5 Discussion and conclusion

Exploiting temporal and spatial variations in rural Chinese residents' food intakes in the CHNS data, our study provides difference-in-difference evidence that rural China's PHI program *can* stimulate food consumption, the most basic type of consumption. As a sort of insurance, health insurance also has the function of reducing uncertainties about one's future well-being and medical spending. Thus, once enrolled, the insured may have the incentive to reduce their precautionary savings, thereby increasing their disposable income. To the extent that food is a “normal” good, the increase in disposable income will induce the insured to consume more of that good. It is also possible that NCMS enrollment stimulates food consumption through knowledge dissemination.^14^ Note that enrollment in the NCMS has been found to increase the frequency of healthcare utilization by the insured (Qin et al., 2014). In the process, the insured may have acquired more knowledge on the health benefits of an improved diet, which in turn induces them to improve their diet structure. The reduction in cooking oil and salt consumption found above is likely due to this effect.

However, not all food-stimulation effects we found are beneficial. In particular, enrollment in the NCMS raised the incidence of over-consumption of grains among the insured; the resulting increases in carbohydrates intake may increase their blood glucose levels, exposing them to the risk of type-2 diabetes. Moreover, the magnitude of the beneficial impact appears to be quite modest. For example, given the average DBI-U score of -34 (“modest” under-consumption) among NCMS participants at the baseline, enrollment in the NCMS pushes their diet balance toward the level of “low” under-consumption (with a DBI-U score between -25 and -13) by roughly one-sixth of the distance between “low” and “modest” under-consumption (9 points). Clearly, other measures may be needed to further improve rural Chinese residents’ diet structure.

Before closing, we note two limitations of this study. First, due to data limitations, we are unable to construct indicators that reflect all aspects of one's diet quality. In particular, the CHNS data contain information only on the *amount* of a food item consumed (e.g., beef) but no information on its *quality* (e.g., whether being branded). Second, also due to data limitations, we only managed to evaluate the impact of NCMS enrollment at the program's rollout stage. But as time went by, more features were built into the program. For example, in August 2012, China began to expand the coverage of the NCMS to include the treatment of critical illnesses (Zhao, 2019). Yet, by the time of writing, the 2015 wave of the CHNS has not been fully released, which prevents us from examining the effect of new features of the program.

Nonetheless, we believe that our analyses have provided valuable information on the impact of PHI programs on diet structure and the relative performance of commonly-used diet-structure indicators, which helps inform health and food policy in China and other developing countries.

## Declarations

### Author contribution statement

Qihui Chen: Conceived and designed the experiments; Performed the experiments; Analyzed and interpreted the data; Wrote the paper.

Chunchen Pei: Performed the experiments; Analyzed and interpreted the data; Contributed reagents, materials, analysis tools or data; Wrote the paper.

Juerong Huang Analyzed and interpreted the data; Contributed reagents, materials, analysis tools or data; Wrote the paper.

Guoqiang Tian: Analyzed and interpreted the data; Contributed reagents, materials, analysis tools or data.

### Funding statement

This work was supported by the 2115 Talent Development Program of 10.13039/501100002365China Agricultural University, Financial supports from the National 10.13039/501100001809Natural Science Foundation of China (grant number: 71973134) and the Social Science Foundation of Beijing Municipality (grant number: 19JDYJB029) are gratefully acknowledged.

### Data availability statement

Data associated with this study has been deposited under the url: https://www.cpc.unc.edu/projects/china/data/datasets.

### Declaration of interests statement

The authors declare no conflict of interest.

### Additional information

No additional information is available for this paper.

## Footnotes

1 The central government contributes 10 yuan (≈1.2 U.S. dollars in 2003) per enrollee, provided that local governments and enrollees contribute no less than 10 yuan. The program has been later expanded. In 2009, the reimburse rate was raised. In August 2012, its coverage was expanded to include the treatment of critical illnesses.

2 For example, from 1992 to 2012, daily consumption of salted vegetables per person in rural China dropped from 10.8 g to 3.1 g, that of grains dropped from 593.8 g to 433.5 g, that of livestock and poultry products increased from 37.6 g to 87.2 g, and that of milk and dairy products increased from 3.8 g to 12.1 g (National Health and Family Planning Commission of the People's Republic of China, 2017).

3 At the time of writing, there have been 1,828 research papers and reports that made use of the CHNS data. Source: (https://www.cpc.unc.edu/projects/china/publications?b_start:int=0 (accessed on January 25, 2022).

4 Although the 2016 version of the *Guidelines* is available, we opt to use the 2007 version. This is because the guidelines were established with reference to the *current* level of living standards, and the 2007 version matches more closely the situation of our sample individuals.

5 Note that while the official definition of the DDS requires data on one's food intake on only one day (24 h), the CHNS provides daily food-intake data for three consecutive days for each sample individual. With these data, we first calculated the DDS for each day and then averaged the scores over three days. The three-day averaging helps eliminate daily fluctuations in one's food intakes, thereby providing a more accurate measure of his/her usual dietary structure.

6 Since the CHNS did not collect detailed information on individuals' drinking water, we can use only six out of seven components to construct the DBI (Table 2, column 2). Yet, drinking water is unlikely to be affected by PHI programs' consumption-stimulation function. Thus, it seems safe to leave it out in the construction of the DBI.

7 Note that there are two non-participants groups. The first consists of non-participants in counties where the NCMS had been implemented. The second group consists of individuals in counties where the NCMS had not yet been implemented by 2006. Bai and Wu (2014) and Chen et al. (2020) reported results obtained using the two groups as separate control groups, which are comparable. In this study, estimations using either control group also yield quite comparable results; we thus opt to pool the two non-participants groups together as a single control group to increase sample size. Results obtained using the two groups as separate control groups are available upon request.

8 We use only baseline characteristics to estimate propensity scores to circumvent potential reverse causality from NCMS participation on some variables (e.g., income and working status) in $X_{it}$.

9 Results reported in Table 3 show that the two approaches yielded very comparable estimates.

10 As explained above, the estimation of propensity scores does not include community FEs. This is because for communities that had not been covered in the NCMS, community FEs would predict a resident's enrollment status perfectly, leaving no room for other predictors to play a role. Thus, we left out community FEs in the estimation of propensity scores. Yet as seen from Table 3, the results are comparable across specifications.

11 Note that the DDS also fails to capture the effects of gender, marital status and household configuration.

12 The set of nutrition-intakes variables have been examined by Ma and Zhang (2011) and Liu (2016). We do not report the results of testing the parallel-trend assumption for those variables here to avoid redundancy. Results are available upon request.

13 We thank an anonymous reviewer for pointing out this issue.

14 We thank an anonymous reviewer for suggesting this potential channel. Unfortunately, the CHNS data lack the information needed to pin down the exact working channels.
